# Integrative microRNAome analysis of skeletal muscle of *Colossoma macropomum* (tambaqui), *Piaractus mesopotamicus* (pacu), and the hybrid tambacu, based on next-generation sequencing data

**DOI:** 10.1186/s12864-021-07513-5

**Published:** 2021-04-06

**Authors:** Bruno E. A. Fantinatti, Erika S. Perez, Bruna T. T. Zanella, Jéssica S. Valente, Tassiana G. de Paula, Edson A. Mareco, Robson F. Carvalho, Silvano Piazza, Michela A. Denti, Maeli Dal-Pai-Silva

**Affiliations:** 1 Department of Structural and Functional Biology, Institute of Biosciences, São Paulo State University – UNESP, Botucatu, Sao Paulo 18618-970 Brazil; 2 Ninth of July University – UNINOVE, Bauru, Sao Paulo Brazil; 3 Department of Cellular, Computational and Integrative Biology – CIBIO, University of Trento, Trento, Italy; 4 University of Western Sao Paulo – UNOESTE, Presidente Prudente, Sao Paulo Brazil

**Keywords:** miRNAoma, Skeletal muscle, Fish

## Abstract

**Background:**

*Colossoma macropomum* (tambaqui) and *Piaractus mesopotamicus* (pacu) are good fish species for aquaculture. The tambacu, individuals originating from the induced hybridization of the female tambaqui with the male pacu, present rapid growth and robustness, characteristics which have made the tambacu a good choice for Brazilian fish farms. Here, we used small RNA sequencing to examine global miRNA expression in the genotypes pacu (PC), tambaqui (TQ), and hybrid tambacu (TC), (Juveniles, *n* = 5 per genotype), to better understand the relationship between tambacu and its parental species, and also to clarify the mechanisms involved in tambacu muscle growth and maintenance based on miRNAs expression.

**Results:**

Regarding differentially expressed (DE) miRNAs between the three genotypes, we observed 8 upregulated and 7 downregulated miRNAs considering TC vs. PC; 14 miRNAs were upregulated and 10 were downregulated considering TC vs. TQ, and 15 miRNAs upregulated and 9 were downregulated considering PC vs. TQ. The majority of the miRNAs showed specific regulation for each genotype pair, and no miRNA were shared between the 3 genotype pairs, in both up- and down-regulated miRNAs. Considering only the miRNAs with validated target genes, we observed the miRNAs miR-144-3p, miR-138-5p, miR-206-3p, and miR-499-5p. GO enrichment analysis showed that the main target genes for these miRNAs were grouped in pathways related to oxygen homeostasis, blood vessel modulation, and oxidative metabolism.

**Conclusions:**

Our global miRNA analysis provided interesting DE miRNAs in the skeletal muscle of pacu, tambaqui, and the hybrid tambacu. In addition, in the hybrid tambacu, we identified some miRNAs controlling important molecular muscle markers that could be relevant for the farming maximization.

**Supplementary Information:**

The online version contains supplementary material available at 10.1186/s12864-021-07513-5.

## Background

*Colossoma macropomum* (tambaqui) and *Piaractus mesopotamicus* (pacu) are fish species that have widely accepted in the consumer market. In addition, they present desirable characteristics for an intensive breeding environment as rapid growth and optimal adaptation to artificial feeding [1].

Noncoding RNAs have become a very important tool for carrying out different types of evolutionary and expression profile experiments. In such way, many databases and protocols for annotation have been developed [2–4].

It is known that Hybrid individuals possess desirable characteristics for production, such as high growth rate, higher resistance to disease, and higher quality of meat [5]. For this reason, several producers have chosen to cultivate the hybrid between the induced crossing of the tambaqui female and the pacu male, the tambacu. Tambacu, in spite of the few genetic information, has the capacity to be more resistant to parasites and stress [6]. Furthermore, tambacu is considered a fish with great potential for Brazilian aquaculture, since it presents a high and fast growth rate, including the skeletal muscle and higher resistance to low temperatures, which contribute to increasing their rusticity [7], thus representing an important model to study. The skeletal muscle is directly involved in the growth of the fish, corresponding to about 35–60% of the body weight of the animal [8]. This abundant muscle mass enables the survival of the animals in the aquatic environment and is commercially important for animal production, since it is one of the most important food sources for human diet [9].

With the focus of massive data analysis, non-coding RNAs have been highly explored in recent years considering their strong impact on controlling several biological processes [10, 11]. Among non-coding RNAs, miRNAs (miRNAs), a well reported class of small non-coding RNAs, are known to perform a fine regulation of gene activity in a post-transcriptional mode upon their association with RNA-induced silencing complex (RISC) and binding via base-complementarity to target mRNAs, resulting in their translational repression and/or in their degradation [12, 13].

Some miRNAs are known to be specifically or highly expressed in the cardiac and/or skeletal muscles and have been dubbed “myo-miRs” [14]. In fish, recent studies have brought information regarding the evolution and genomic organization of myo-miRNAs [15].

Considering the important role of miRNAs in cell physiology, the aim of our study was to evaluate the global miRNA profile in skeletal muscle of pacu, tambaqui, and the hybrid tambacu, to investigate differentially expressed miRNAs and the ontology analysis considering the comparison between the hybrid with and its parental genotypes, to better understand the molecular signaling pathways involved in tambacu muscle development, growth, and maintenance. In this way, the present work can be an important source of information to supports studies that address the advantages of adopting hybrids for cultivation, which are still scarce [7].

## Results

Early juvenile fish *Colossoma macropomum*, *Piaractus mesopotamicus*, and hybrid tambacu were studied. All the extracted RNA samples were analyzed by NanoVue (GE Healthcare), and Bioanalyzer (Agilent Technologies) and only samples with a RIN ≥ 8 were selected for the sequencing procedures.

**Fig. 1:**
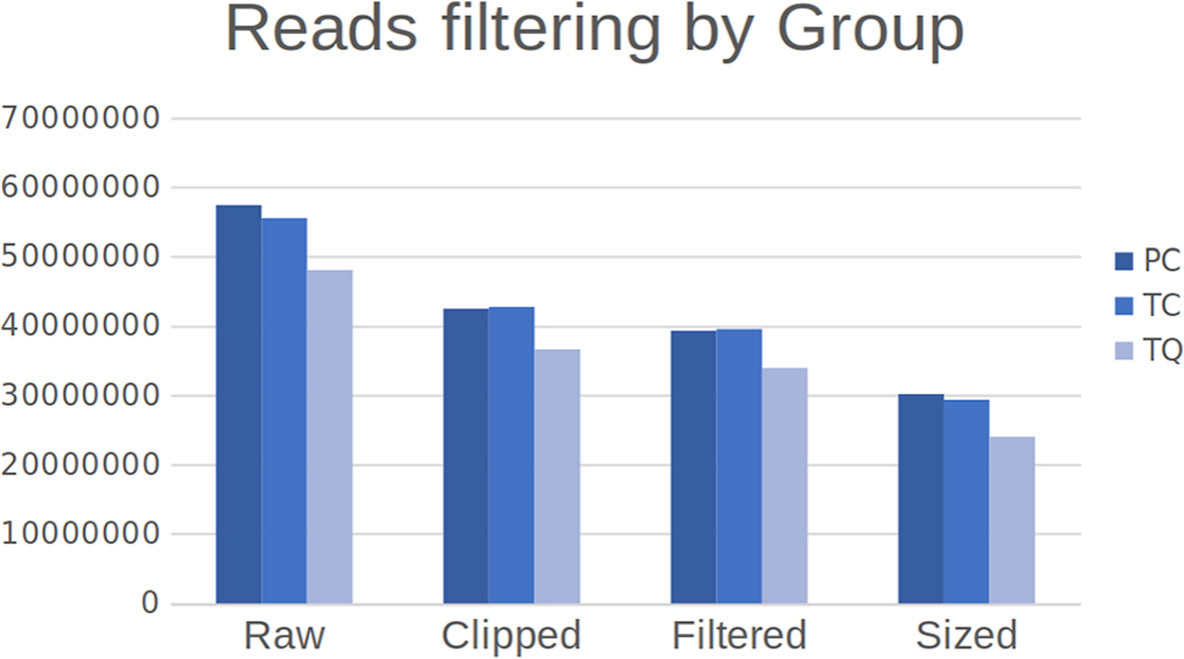
Bar graphs showing the read numbers throughout the filtering process. Raw (raw data after downloading), Clipped (remaining reads after removing adaptors and reads shorter than 17 nt), Filtered (remaining reads after filtering by read quality) and Sized (remaining reads after removing sequences longer than 26 nt)

**Fig. 2:**
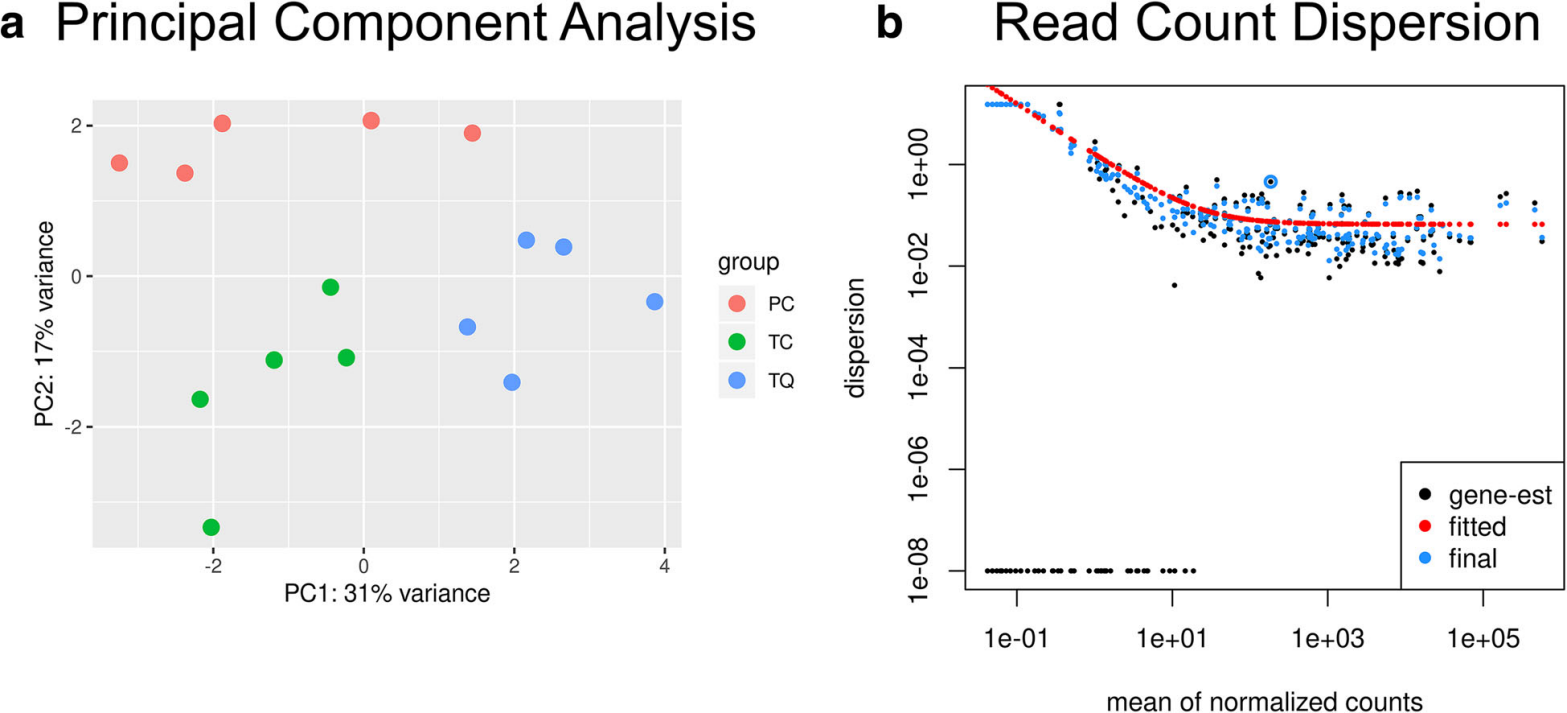
**a**: Principal Component Analysis obtained using the full log-transformed list of mean read-count miRNAs showing the different grouping samples. **b**: Dispersion Plot showing high dispersion values below a mean count of 10

After preprocessing steps, the number of reads decreased to approximately 50% due to the removal of low-quality reads and the adapters, and the removal of sequences not matching the minimum/maximum size corresponding to miRNAs (Fig. 1, Table 1, and Additional file 1). We obtained a total alignment average of 51,78%, considering all samples analyzed. As a result of featureCounts processing (see Additional files 2 and 3), a count matrix was generated for each miRNA present in the reference (see Additional file 4). As part of the pipeline, Principal Component Analysis (PCA) (Fig. 2a) and Dispersion Analysis (Fig. 2b) were run to cluster the samples based on the expression values and to observe the quality of the data. We observed the samples separated into clusters among samples, indicating that the different groups really correspond to different genotypes. Also, the Dispersion Analysis showed that below a mean read count of 10, the dispersion of the data increased dramatically. Thus, miRNAs presenting a mean read count < 10 were filtered out to keep only miRNAs not presenting high dispersion levels.

**Table 1:** Total read numbers throughout the filtering process. Raw (raw data after downloading), Clipped (remaining reads after removing adaptors and reads shorter than 17 nt), Filtered (remaining reads after filtering by read quality) and Sized (remaining reads after removing sequences longer than 26 nt)

Samples	Counts				Overall Remaining (%)
	Raw	Clipped	Filtered	Sized	
PC	57,376,715	42,480,101	39,296,309	30,132,774	52,52
TC	55,492,134	42,720,612	39,513,638	29,310,231	52,82
TQ	48,058,874	36,611,767	33,906,594	24,038,384	50,02
					**51,78**

**Fig. 3:**
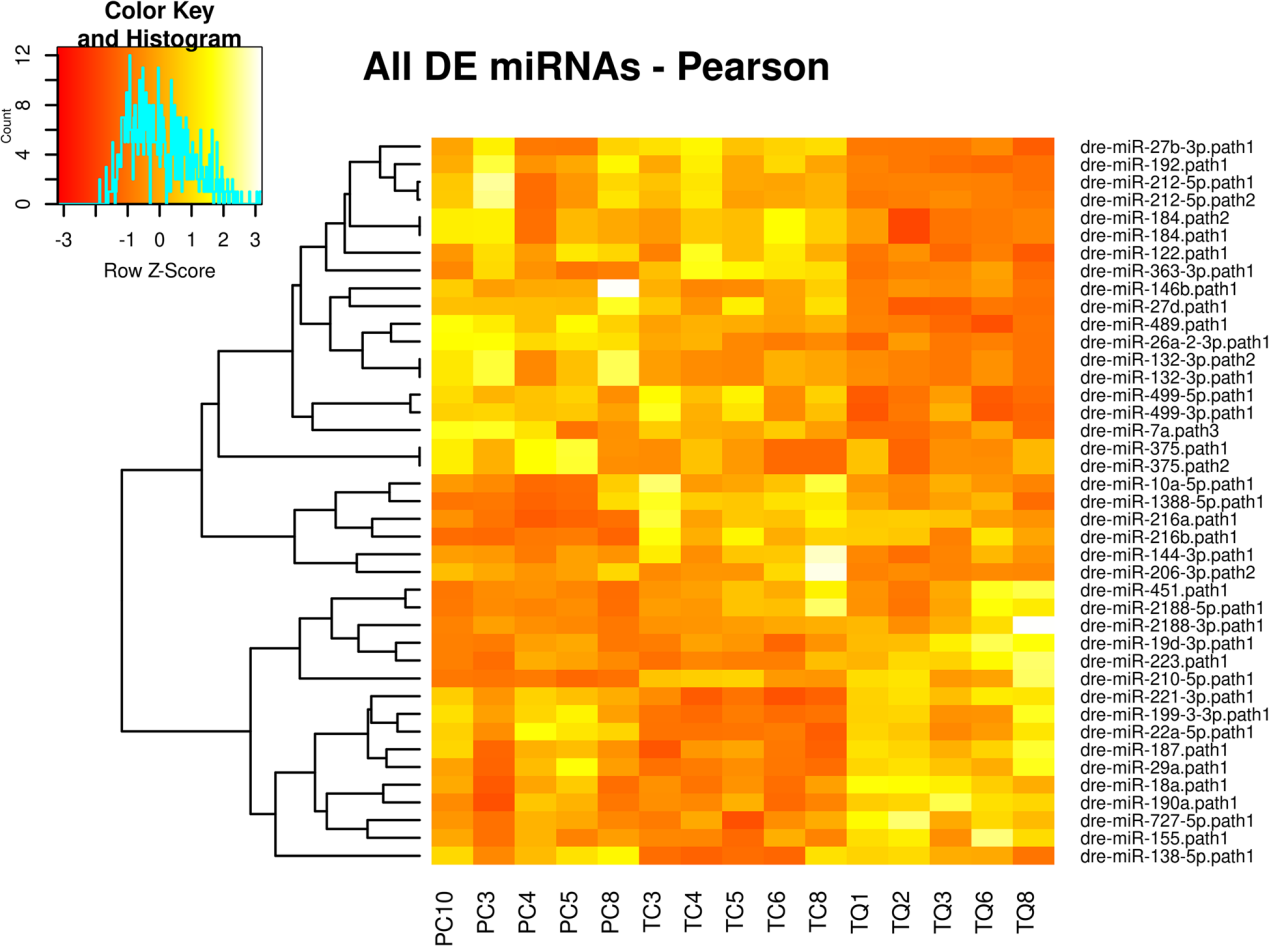
Row-clusterized heatmap showing differentially expressed miRNAs between all groups. Light colors, high expressed. Dark colors, low expressed. Rows were clusterized using Pearson correlation

**Fig. 4:**
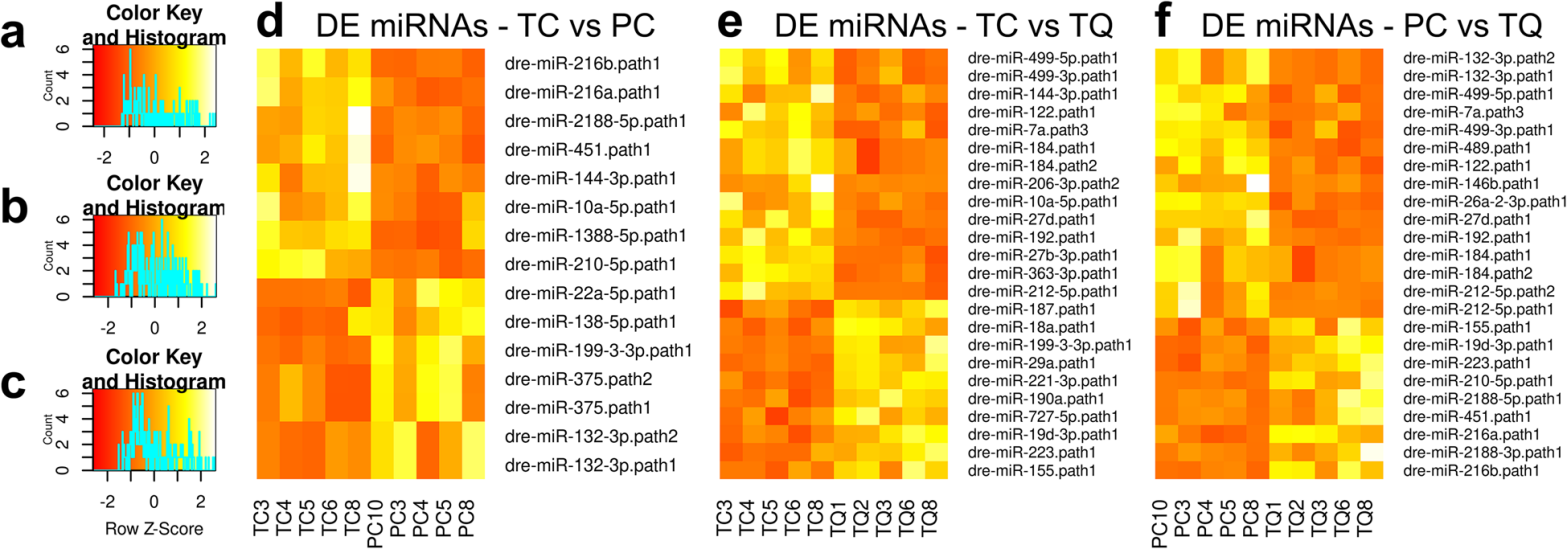
Non-clusterized heatmap showing differentially expressed miRNAs between groups. TC vs. PC (**a** and **d**), TC vs. TQ (**b** and **e**), and PC vs. TQ (**c** and **f**). Light colors, high expressed. Dark colors, low expressed. miRNAs have been ordered according to a decreasing Log2FC

**Fig. 5:**
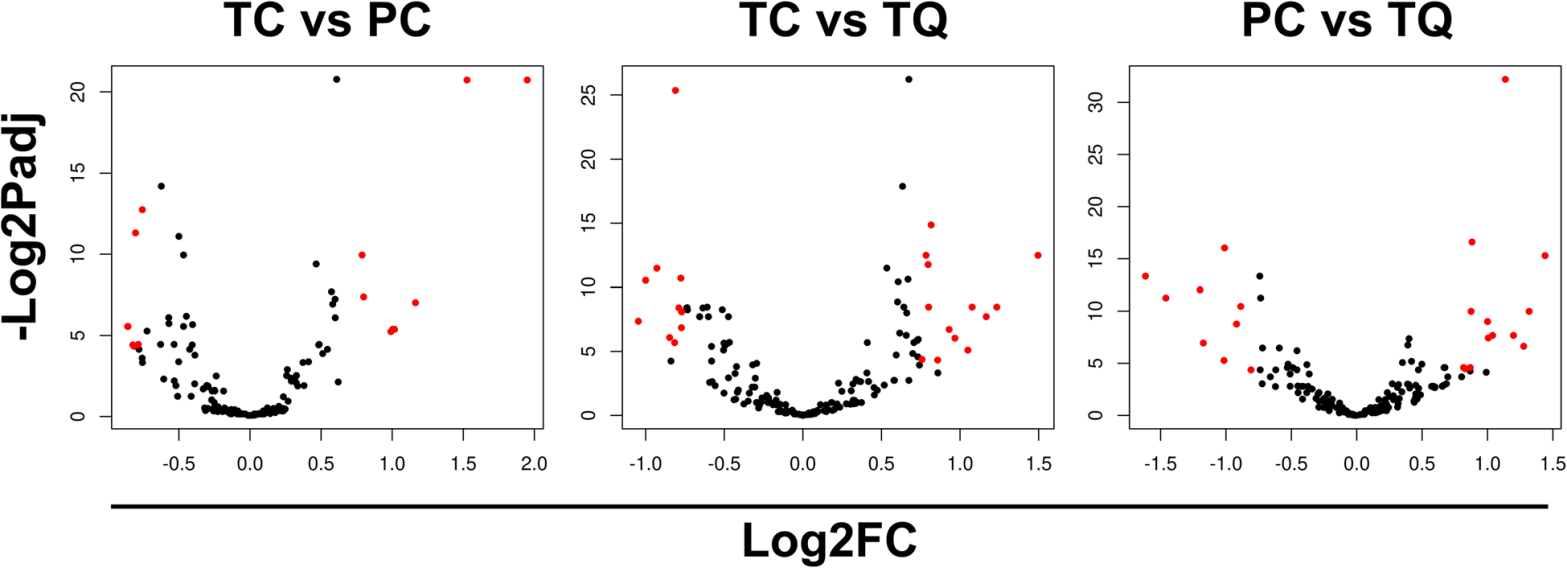
Volcano Plots showing differentially expressed miRNAs between the comparisons. Log2FC ≥ 0.75 and padj ≤0.05. X axis, Log2FC. Y axis, −Log2padj. Differentially expressed miRNAs (DE miRNAs) are shown in red color

Differentially expressed (DE) miRNAs groups were observed in a row-clustered heatmap containing all the comparison genotype pairs (TC vs. PC, TC vs. TQ, and PC vs. TQ) and all the miRNAs detected as DE (Fig. 3). Based on color histogram, it was possible to observe the variations in terms of expression levels between up-regulated and down-regulated miRNAs throughout the three genotypes analyzed. A relative high number of DE miRNAs are observed in the data. It Is possible to detect such a balance of DE miRNAs, where there is no comparison only with up- or down-regulated miRNAs (Figs. 4 and 5).

DE analysis showed 8 upregulated and 7 downregulated miRNAs for TC vs. PC genotype pair (Fig. 4a and d, Table 2 and Additional file 5), 14 upregulated and 10 downregulated miRNAs for TC vs. TQ genotype pair (Fig. 4b and e, Table 3 and Additional file 6), and 15 upregulated and 9 downregulated miRNAs for the PC vs. TQ genotype pair (Fig. 4c and f, Table 4 and Additional file 7). A full list exclusive for DE miRNAs can be observed on Additional file 8.

**Table 2:** DE miRNAs (TC vs. PC). Up-regulated and down-regulated miRNAs are in bold and italic, respectively, followed by log2FC and padj values. miRNAs with experimentally validated interactions are underlined

miRNA (TC vs PC)	log2FoldChange	padj
**dre-miR-216b.path1**	**1.95011465502623**	**5,75E+ 07**
**dre-miR-216a.path1**	**1.52622128306155**	**5,75E+ 07**
**dre-miR-2188-5p.path1**	**1.16367923053015**	**0.00768680121745896**
**dre-miR-451.path1**	**1.01615109227537**	**0.0239688647031353**
**dre-miR-144-3p.path1**	**1.00354412348832**	**0.023884645507697**
**dre-miR-10a-5p.path1**	**0.990777104704709**	**0.0264397675354512**
**dre-miR-1388-5p.path1**	**0.799356542997587**	**0.00599026348294433**
**dre-miR-210-5p.path1**	**0.788278489052125**	**0.00101236465373185**
*dre-miR-22a-5p.path1*	*−0.757442401072745*	*0.000145626758222292*
*dre-miR-138-5p.path1*	*−0.785285789697823*	*0.045680698066672*
*dre-miR-199-3-3p.path1*	*−0.805140039326324*	*0.000392850813725473*
*dre-miR-375.path2*	*−0.814180847823428*	*0.0496601836495912*
*dre-miR-375.path1*	*−0.824148790777728*	*0.0467129686944908*
*dre-miR-132-3p.path2*	*−0.85811978487659*	*0.0212262367860986*
*dre-miR-132-3p.path1*	*−0.860515272540314*	*0.0212262367860986*

**Table 3:** DE miRNAs (TC vs. TQ). Up-regulated and down-regulated miRNAs are in bold and italic, respectively, followed by log2FC and padj values. miRNAs with experimentally validated interactions are underlined

miRNA (TC vs TQ)	log2FoldChange	padj
**dre-miR-499-5p.path1**	**1.49580994425598**	**0.000173839481127439**
**dre-miR-499-3p.path1**	**1.23356812765229**	**0.00282946156806081**
**dre-miR-144-3p.path1**	**1.16659137509147**	**0.00474941074241266**
**dre-miR-122.path1**	**1.07694111084953**	**0.00282946156806081**
**dre-miR-7a.path3**	**1.04984406425985**	**0.0290652273981748**
**dre-miR-184.path1**	**0.967557360144771**	**0.0152050542458195**
**dre-miR-184.path2**	**0.967557360144771**	**0.0152050542458195**
**dre-miR-206-3p.path2**	**0.930801815773025**	**0.0094757292155134**
**dre-miR-10a-5p.path1**	**0.857505087018255**	**0.0499676766826888**
**dre-miR-27d.path1**	**0.816105786892291**	**3,36E+ 09**
**dre-miR-192.path1**	**0.800332526539842**	**0.00282946156806081**
**dre-miR-27b-3p.path1**	**0.796942141264382**	**0.000287445330003991**
**dre-miR-363-3p.path1**	**0.783911546005418**	**0.000173839481127439**
**dre-miR-212-5p.path1**	**0.758083013615199**	**0.0481768505247122**
*dre-miR-187.path1*	*−0.771077479498364*	*0.0036724290357109*
*dre-miR-18a.path1*	*−0.772496606919669*	*0.00861750612968111*
*dre-miR-199-3-3p.path1*	*−0.776134338005323*	*0.000598753742363573*
*dre-miR-29a.path1*	*−0.788357562619249*	*0.00295632195436478*
*dre-miR-221-3p.path1*	*−0.810494830489444*	*2,34E+ 06*
*dre-miR-190a.path1*	*−0.815779490936263*	*0.0193213894766877*
*dre-miR-727-5p.path1*	*−0.847542177958638*	*0.0147146666881142*
*dre-miR-19d-3p.path1*	*−0.928688683649729*	*0.000349643237371769*
*dre-miR-223.path1*	*−1.00025248763242*	*0.000674009078964609*
*dre-miR-155.path1*	*−1.0467224627299*	*0.00608254996948173*

**Table 4:** DE miRNAs (PC vs. TQ). Up-regulated and down-regulated miRNAs are in bold and italic, respectively, followed by log2FC and padj values. miRNAs with experimentally validated interactions are underlined

miRNA (PC vs TQ)	log2FoldChange	padj
**dre-miR-132-3p.path2**	**1.44051489782147**	**2,49E+ 08**
**dre-miR-132-3p.path1**	**1.44002708507766**	**2,49E+ 08**
**dre-miR-499-5p.path1**	**1.31828295307616**	**0.000989191572851596**
**dre-miR-7a.path3**	**1.27678095695125**	**0.0100716056640765**
**dre-miR-499-3p.path1**	**1.19850803219891**	**0.00488526006580054**
**dre-miR-489.path1**	**1.1362366110581**	**2,06E+ 04**
**dre-miR-122.path1**	**1.03864026089626**	**0.00488526006580054**
**dre-miR-146b.path1**	**1.00598270712002**	**0.00577894745989986**
**dre-miR-26a-2-3p.path1**	**1.00052719757472**	**0.00194408881995706**
**dre-miR-27d.path1**	**0.881725080253345**	**1,00E+ 09**
**dre-miR-192.path1**	**0.8745609159566**	**0.000989191572851596**
**dre-miR-184.path1**	**0.867821451883665**	**0.0414916261017677**
**dre-miR-184.path2**	**0.867821451883665**	**0.0414916261017677**
**dre-miR-212-5p.path2**	**0.83092978326804**	**0.0450498513190579**
**dre-miR-212-5p.path1**	**0.817076572301239**	**0.0414916261017677**
*dre-miR-155.path1*	*−0.808575426957622*	*0.0483381089257489*
*dre-miR-19d-3p.path1*	*−0.886901002648801*	*0.000711319019021908*
*dre-miR-223.path1*	*−0.918993216115796*	*0.00229667926127593*
*dre-miR-210-5p.path1*	*−1.01067899424562*	*1,48E+ 09*
*dre-miR-2188-5p.path1*	*−1.01503627156949*	*0.0258798964614143*
*dre-miR-451.path1*	*−1.17281189217678*	*0.00812402676379883*
*dre-miR-216a.path1*	*−1.19826926904911*	*0.000237764301717364*
*dre-miR-2188-3p.path1*	*−1.45957650464932*	*0.000407849519588126*
*dre-miR-216b.path1*	*−1.61565155335869*	*9,71E+ 09*

**Fig. 6:**
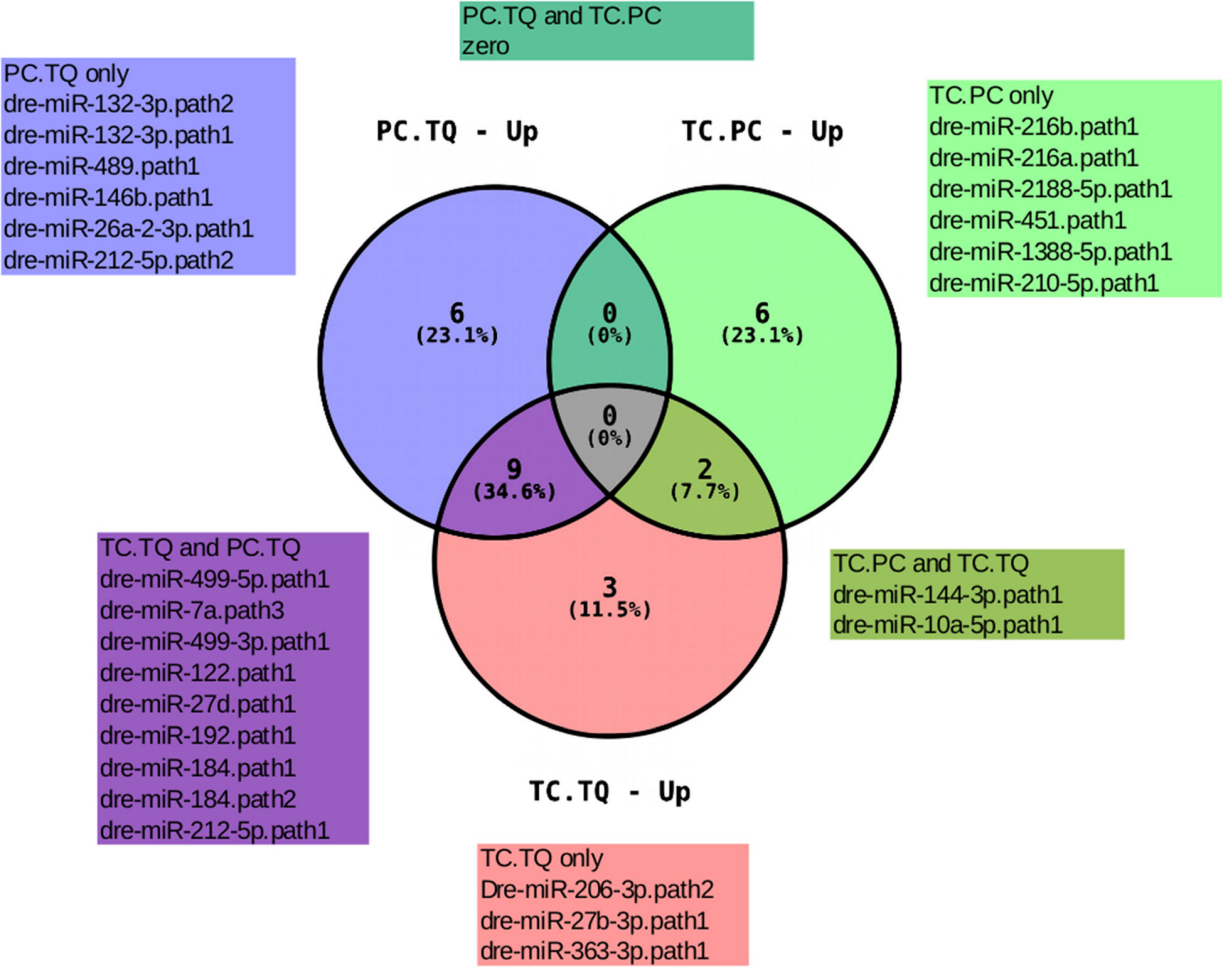
Venn Diagram showing the relationship between up-regulated miRNAs throughout the different groups. The number marked in the overlapping areas shows the common DE miRNAs

**Fig. 7:**
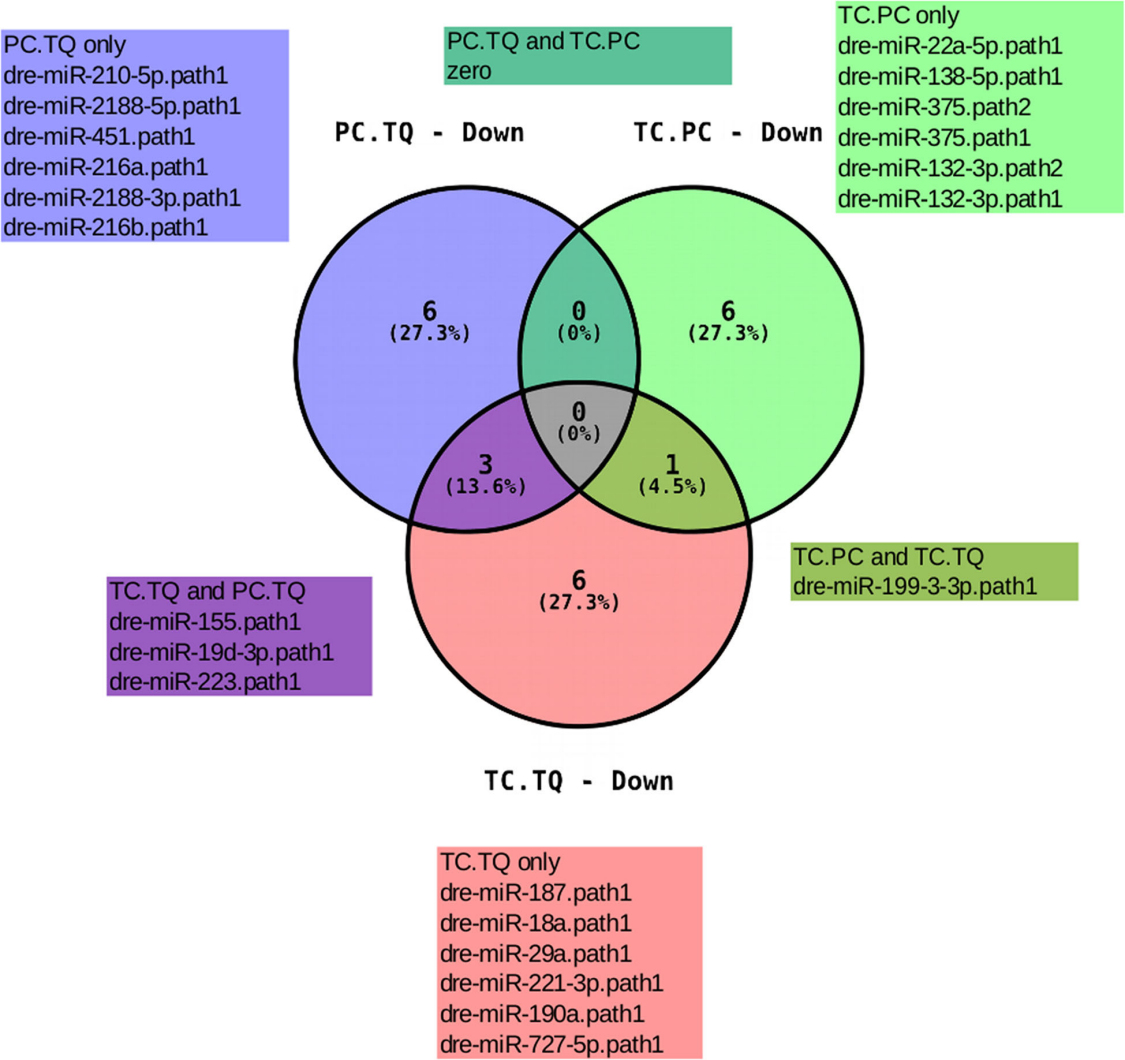
Venn Diagram showing the relationship between down-regulated miRNAs throughout the different groups. The number marked in the overlapping areas shows the common DE miRNAs

**Fig. 8:**
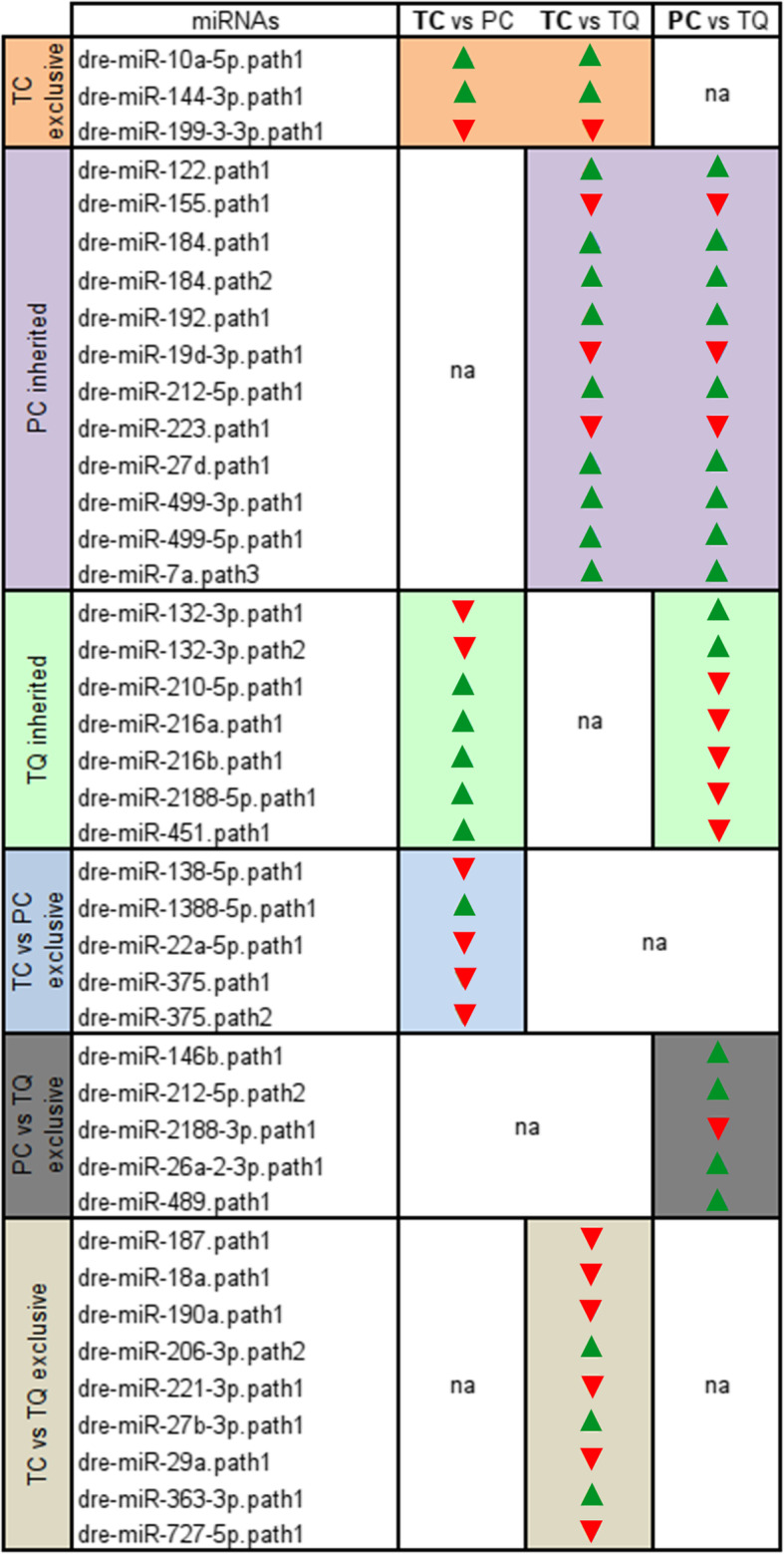
miRNA profiles summary between species. ▲ and ▼: Up- and Down-regulated, respectively. Na: not applicable. MiRNAs were added to six different categories based on their differential expression characteristics. If a given miRNA appears up- or down-regulated only in the hybrid, it presents a hybrid-exclusive behavior. If a given miRNA is up- or down-regulated in the one parental and in the hybrid, it presents a behavior inherited from that parental

According to the Venn diagram data (Figs. 6 and 7), we analyzed the miRNA distribution pattern between the parental species and the hybrid, and it was possible to observe some DE miRNA-sharing between the different genotype’s pairs. For example, considering the upregulated set of miRNAs, miR-144-3p and miR-10a-5p appeared to be upregulated in TC compared to both PC and TQ (Figs. 6 and 8). This leads us to think that the upregulation of these miRNAs could be a result of a multifactorial characteristic involving both profiles given that such miRNAs are upregulated only when the contrast involves the hybrid and such miRNAs are not DE between parental species. A similar characteristic was observed in the down-regulated miRNAs, where miR-199-3-3p appears downregulated in TC when compared with both PC and TQ. These miRNAs are considered as TC-exclusive (in terms of expression levels), as they appear to be up- or down-regulated only in the hybrid compared to the parental species, but not between parental species (Fig. 8). Also, we detected some up- or down-regulated miRNAs only in the hybrid, representing a specific characteristic of this genotype.

We then assigned different miRNAs in 6 different categories regarding inheritance characteristics related to expression levels, i.e., (i) TC-exclusive (for those miRNAs differentially expressed only in TC compared with both parental species), (ii) PC-inherited (for those miRNAs with expression patterns similar to PC expression levels), (iii) TQ-inherited (for those miRNAs with expression patterns similar to TQ expression levels), (iv) TC vs. PC-exclusive (for miRNAs that are differentially expressed only when TC and PC are involved in comparisons), (v) PC vs. TQ-exclusive (for miRNAs that are differentially expressed only when PC and TQ are involved in comparisons), and (vi) TC vs. TQ-exclusive (for miRNAs that are differentially expressed only when TC and TQ are involved in comparisons). miRNAs are also discriminated in up- (▲) and downregulated (▼) (Fig. 8).

**Fig. 9:**
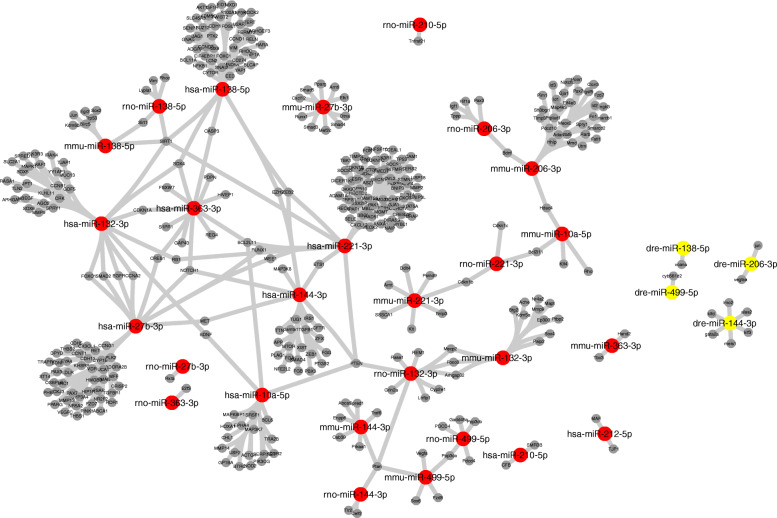
Experimentally validated interactions among DE miRNAs based on *Danio rerio*, *Homo sapiens*, *Mus musculus* and *Rattus novergicus*. *Danio rerio* interaction data is shown in yellow nodes

**Fig. 10:**
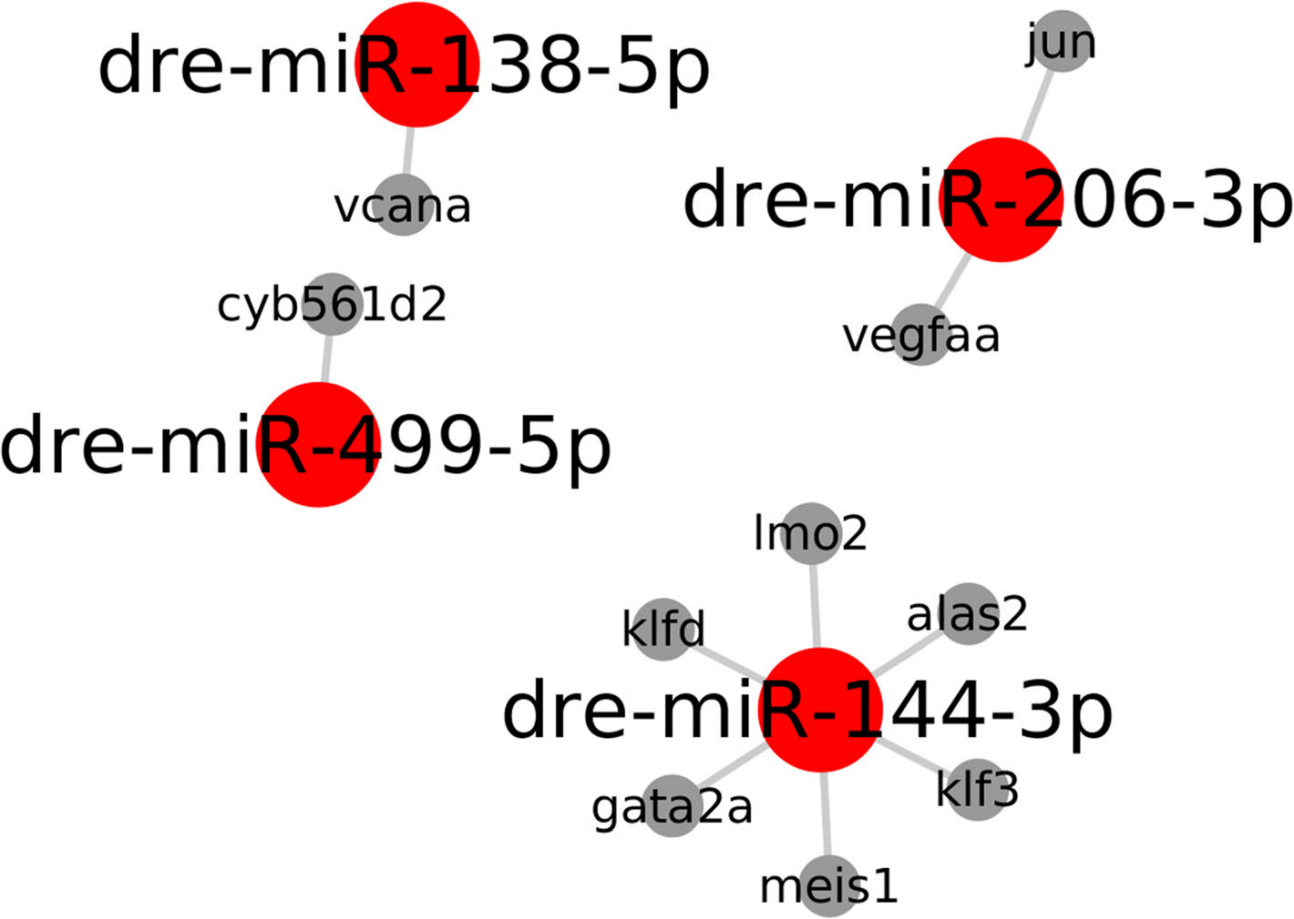
Experimentally validated interactions among DE miRNAs based on *Danio rerio*. *vcana* - Versican a; *cyb561d2* - Cytochrome B561 Family Member D2; *jun* - AP-1 Transcription factor Subunit; *vegfa* - Vascular Endotelial Growth Factor A; *lmo2* - LIM Domain Only 2; *klfd* - Kruppel-like factor d; *gata2a* - GATA-binding protein 2; *meis1* - Meis Homeobox 1; *klf3* - Kruppel Like Factor 3; *alas2*–5-aminolevulinate synthase 2; *hif1* - Hypoxia-Inducible Factor 1

In the search for target genes involved in DE miRNAs expression, we searched for experimentally validated targets using miRTarBase dataset version 7 [16]. Two networks comprising only validated data were detected (Figs. 9 and 10). A network presenting strong validated interactions between target genes and DE miRNAs expression for *Homo sapiens*, *Mus musculus*, *Rattus norvegicus*, and *Danio rerio* are presented (Fig. 9). We observed interactions among 31 known miRNAs, and the top five miRNAs with a higher number of interactions were hsa-miR-221-3p, has-miR-27b-3p, hsa-miR-138-5p, mmu-miR-206-3p, and hsa-miR-132-3p, targeting respectively 72, 52, 47, 32, and 32 target genes. Considering the *Danio rerio* validated interaction data (Fig. 10), we observed that the network presents interactions involving four DE miRNAs and 10 target genes: dre-miR-138-5p (*vcana*), dre-miR-206-3p (*vegfa* and *jun*), dre-miR-499-5p (*cyb561d2*), and dre-miR-144-3p (*lmo2*, *klfd*, *gata2a*, *meis1*, *klf3*, and *alas2*).

**Fig. 11:**
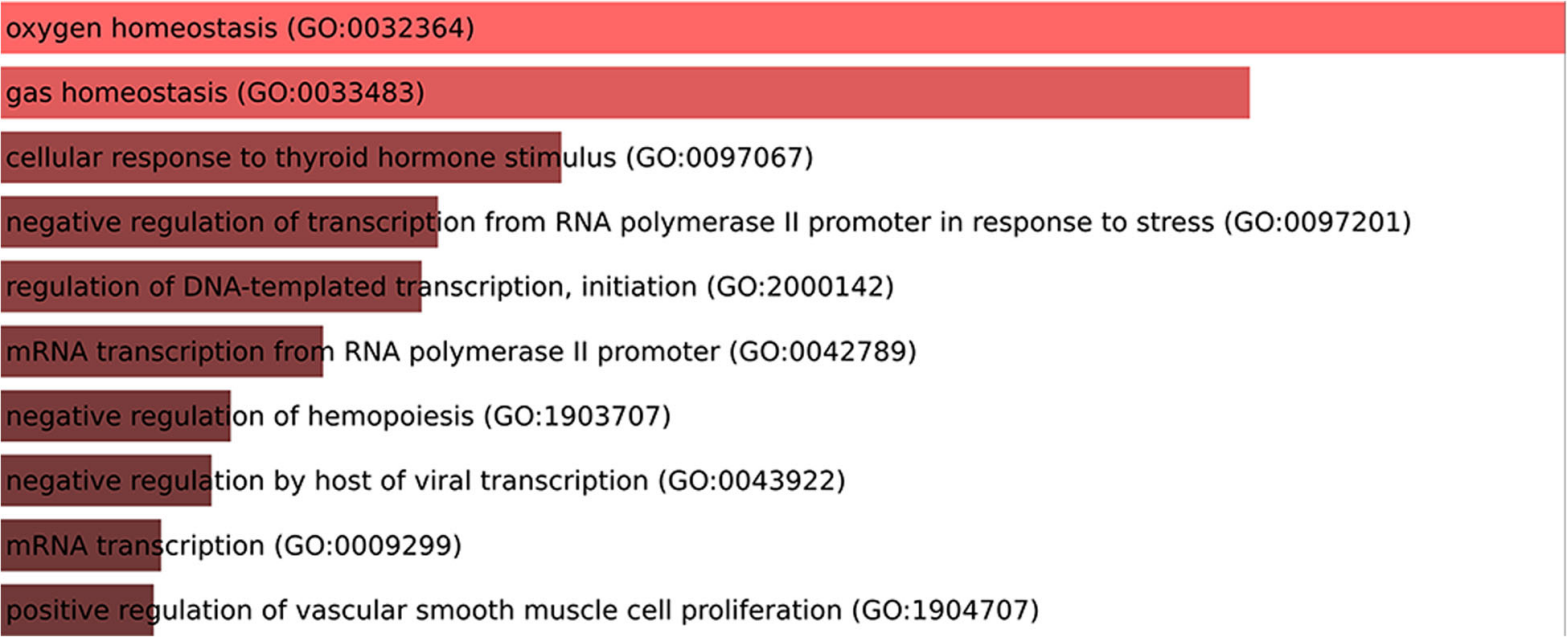
Biological processes involved with validated-target genes of the DE miRNAs. Upper bars represent more significant data. Lower bars represent less significant data

**Fig. 12:**
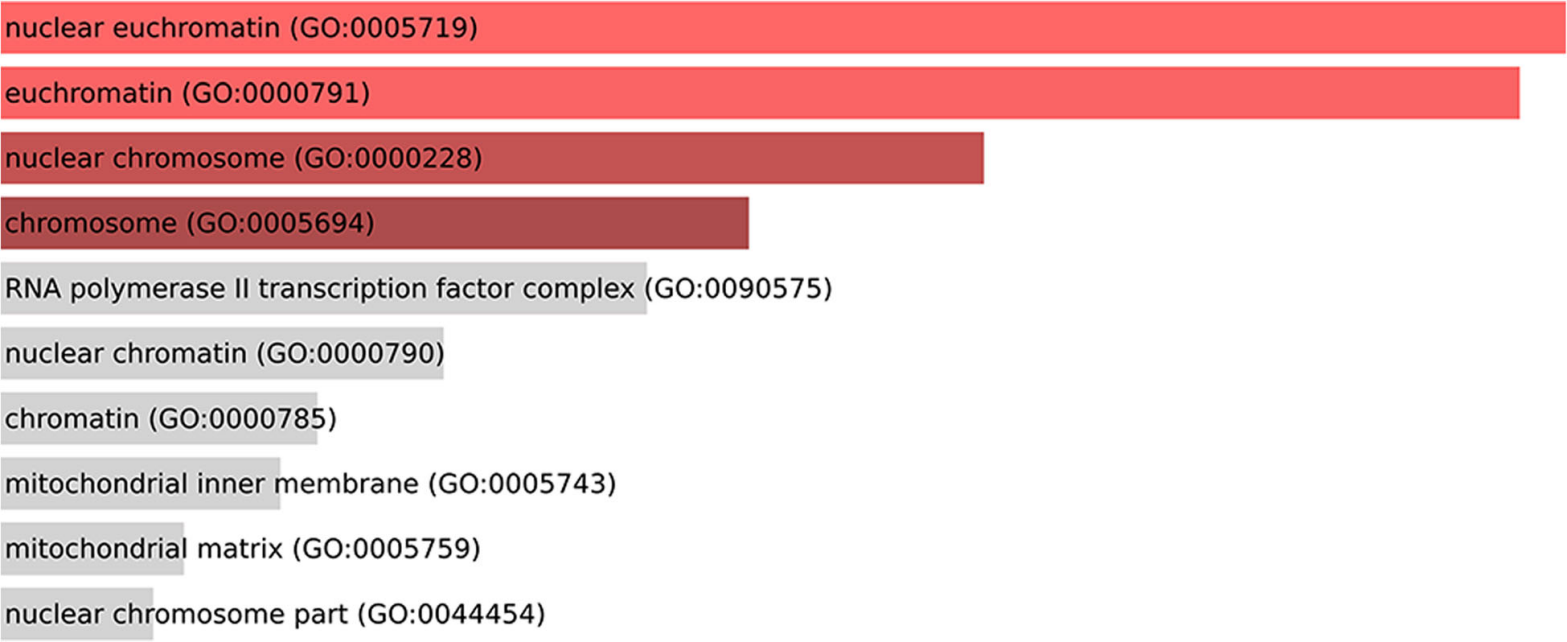
Cellular components involved with validated-target genes of the DE miRNAs. Upper bars represent more significant data. Lower bars represent less significant data

**Fig. 13:**
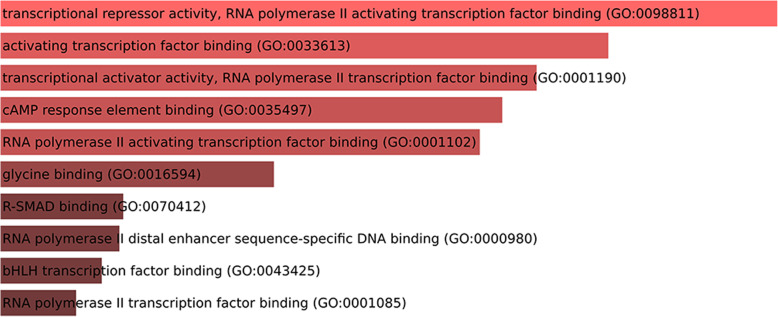
Molecular functions involved with validated-target genes of the DE miRNAs. Upper bars represent more significant data. Lower bars represent less significant data

Ontology analysis carried out by using Enrichr [17], considering the genes validated as targets of the DE miRNAs (considering the *Danio rerio* validated interactions) showed that the interacting-validated genes are involved in various categories of ontology. For Biological Processes, Cellular Components, and Molecular Functions, the top enriched terms were, respectively, oxygen homeostasis (the most representative gene was 5-aminolevulinate synthase 2 - *alas2*), nuclear euchromatin (the most representative gene was *jun*), and transcriptional repressor activity, RNA polymerase II activating transcription factor binding (the most representative genes were *jun* and *lmo2*) (Figs. 11, 12 and 13 and Table 5).

**Table 5:** Ontology analysis by gene-term enrichment, considering the *Danio rerio* interacting-validated genes targets of the DE miRNAs, showing the genes and the ontology categories of signaling pathways. *alas2–*5-aminolevulinate synthase 2; *lmo2* - LIM Domain Only 2; *jun* - AP-1 Transcription factor Subunit; *meis1* - Meis Homeobox 1

	Signaling Pathways	Genes
**Biological Process**	oxygen homeostasis (GO:0032364)	*alas2*
	gas homeostasis (GO:0033483)	*alas2*
	cellular response to thyroid hormone stimulus (GO:0097067)	*lmo2*
	negative regulation of transcription from RNA polymerase II promoter in response to stress (GO:0097201)	*jun*
	regulation of DNA-templated transcription, initiation (GO:2000142)	*jun*
	mRNA transcription from RNA polymerase II promoter (GO:0042789)	*lmo2*
	negative regulation of hemopoiesis (GO:1903707)	*meis1*
	negative regulation by host of viral transcription (GO:0043922)	*jun*
	mRNA transcription (GO:0009299)	*lmo2*
	positive regulation of vascular smooth muscle cell proliferation (GO:1904707)	*jun*
**Cellular Component**	nuclear euchromatin (GO:0005719)	*jun*
	euchromatin (GO:0000791)	*jun*
	nuclear chromosome (GO:0000228)	*jun*
	chromosome (GO:0005694)	*jun*
**Molecular Function**	transcriptional repressor activity, RNA polymerase II activating transcription factor binding (GO:0098811)	*jun*; *lmo2*
	activating transcription factor binding (GO:0033613)	*jun*; *lmo2*
	transcriptional activator activity, RNA polymerase II transcription factor binding (GO:0001190)	*jun*; *lmo2*
	cAMP response element binding (GO:0035497)	*jun*
	RNA polymerase II activating transcription factor binding (GO:0001102)	*jun*; *lmo2*
	glycine binding (GO:0016594)	*alas2*
	R-SMAD binding (GO:0070412)	*jun*
	RNA polymerase II distal enhancer sequence-specific DNA binding (GO:0000980)	*jun*

## Discussion

Next-generation sequencing has been widely applied for global analysis in several studies involving miRNAs and skeletal muscle. Nachtigall et al. employed next-generation sequencing in Nile Tilapia (*Oreochromis niloticus*), to obtain information regarding evolutionary pathways with emphasis on muscle miRNAs [15]. The authors identified that there are large syntenic blocks in the genome, possibly being linked to a common function of such miRNAs, and as well specific role of miR-499 expression [18]. Gomes et al. also used next generation sequencing to analyze samples of liver and skin from tambaqui (*Colossoma macropomum*) to characterize and identify the expression levels of miRNAs and the interaction with target genes having *D. rerio* as reference [19]. The analysis performed showed that although there are tissue-specific miRNAs expression profile, some miRNAs can be shared between different tissues, as liver and skin. The most expressed miRNAs in both tissues enriched signaling pathways that control several biological processes with a large gene network.

Previous studies in our group have also analyzed the expression of some muscle specific miRNAs in the skeletal muscle of fish involving fasting and re-feeding treatments and muscle development [20, 21]. The authors observed that some miRNAs (miR-1, miR-133, miR-155, miR-206, and miR-499) presented a possible role in the regulation of factors related to muscle cell proliferation and differentiation and with muscle performance and metabolism, modulating the rate of protein synthesis and degradation.

In the present study, global analysis of miRNAs in the three genotypes identified a small number of differentially expressed miRNAs considering the comparison between the genotypes pairs. The comparison between tambacu and *P. mesopotamicus* (TC vs. PC) showed 15 DE miRNAs, 8 upregulated and 7 downregulated. Considering the comparison between tambacu and *C.macropomum* (TC vs. TQ), we observed 24 DE miRNAs, 14 upregulated and 10 downregulated, and considering the comparison between *P. mesopotamicus* and *C. macropomum* (PC vs. TQ), we observed 24 DE miRNAs, 15 upregulated and 9 downregulated. Interestingly, the majority of the miRNAs were specific for each genotype pair. No miRNA was shared between the 3 genotype pairs, both up and downregulated. Between the upregulated DE miRNAs, 9 overlapped between PC vs. TQ and TC vs. TQ and 2 overlap between TC vs. PC and TC vs. TQ. Considering the downregulated DE miRNAs, 3 overlap between PC vs. TQ and TC vs. TQ, and only one overlaps between TC vs. PC and TC vs. TQ. No miRNA was differentially expressed between all of the three comparison pairs at the same time. Some miRNAs show characteristics that make it possible to track their inheritance pattern by looking into the different groups. For example, some miRNAs appear to be exclusive for the comparison between TC vs. TQ (miR-187, miR-18a, miR-190a, miR-206-3p, miR-221-3p, miR-27b-3p, miR-29a, miR-363-3p, and miR-727-5p), some of which were exclusive in the comparison between PC vs. TQ (miR-146b, miR-212-5p, miR-2188-3p, miR-26a-2-3p, and miR-489). Interestingly, some miRNAs presented a characteristic of being inherited specifically of one genotype, and also, there were some miRNAs that appeared to be up- or down-regulated only in the hybrid (miR-10a-5p, miR-144-3p, and miR-199-3-3p). These miRNAs presented (up or downregulated) behavior, exclusively in the hybrid, not differentially expressed between the parental genotypes.

Ontology analysis by Enrichr, considering the *Danio rerio* validated interaction data, showed that the experimentally validated target genes of the DE miRNAs were grouped into several Biological Processes, Cellular Components, and Molecular Functions categories. The most enriched processes were oxygen homeostasis, nuclear euchromatin and transcriptional repressor activity, and RNA polymerase II activating transcription factor binding, respectively. The more enriched genes in the signaling pathway involved in these processes were *alas2*, *jun, and lmo2*.

miR-206-3p, identified in the present study, was differentially expressed in the comparison pair TC vs. TQ, targets *jun* and *vegfa* genes. *Jun* is a member of the Activator Protein 1 (AP-1) transcription factor family that regulates cell proliferation and differentiation, apoptosis, cellular migration, inflammation, and cell-cell interaction [22]. On the other hand, *vegfa* is involved in vascular development and new blood vessel formation [23, 24] and stimulates endothelial cell migration by activating AP-1 transcription factor *jun* [25]. Skeletal muscle is the most abundant source of VEGFA [26, 27], and skeletal-muscle-specific VEGFA knockout changed angiogenesis in muscle fibers [28].

As *jun* and *vegfa* genes are targets of miR-206-3p and, as this miRNA was observed to be differentially expressed in the comparison pair TC vs. TQ, this is an indication that this miRNA can represent a remarkable characteristic for the hybrid, since these genes are involved in the maintenance of blood irrigation that, in turn, controls the oxygen rates in the tissues.

The miRNA involved in the control of the *alas2 and lmo2* genes was miR-144-3p. This miRNA appeared upregulated in the comparison pairs TC vs. PC and TC vs. TQ and did not appear in the comparison between the parental genotypes PC vs. TQ.

ALAS2 is one of the isozymes of ALAS (5-aminolevulinate synthase) involved in vertebrate heme biosynthesis. This gene is expressed preferentially in erythroid cell-specific mitochondrial enzymes [29, 30] and catalyzes the biosynthesis of bulk heme for hemoglobin production [31–34]. ALAS2 is also regulated by hypoxia-inducible factor HIF. Khenchaduri and colaborators observed the overexpression of ALAS2 in cardiac myoblasts submitted to chronic hypoxia, with a corresponding increase in cellular heme levels. They concluded that, similar to erythroid cells, ALAS2 is positively regulated by hypoxia in cardiac myoblasts with an increase in heme levels [35].

According to Zhang et al., the upregulation of *alas2* during hypoxia is directly mediated by a transcription factor, hypoxia-inducible factor 1 (Hif1) [36], and an increased *Hif-1* that occurs under low oxygen concentration conditions also promotes the activation of genes involved in hypoxia, such as *vegf* [37].

Moreover, it has been shown that the transcription factor LIM domain only 2 (Lmo2) is required for angiogenesis and is important for angiogenic remodeling of the existing capillary network into mature blood vessels [38–40]. Besides, Lmo2 forms common transcription complexes with GATA-binding protein 2 (GATA2) and cooperatively regulate VEGF-induced angiogenesis [41].

Therefore, it is possible that the interaction between miR-144-3p and *alas2* and *lmo2* genes can regulate important pathways related to muscle vascularization, oxygen homeostasis, and oxidative metabolism. We believe that this is an important characteristic that probably could provide, for the hybrid tambacu, more resistance to environments with lower oxygen content, once this miRNA was upregulated only in the hybrid, in the comparison pairs TC vs. PC and TC vs. TQ.

In our study, miR-144-3p was identified as DE in TC vs. PC and TC vs. TQ, and the miR-206-3p described as DE only in the comparison pair TC vs. TQ demonstrated that these miRNAs may represent an important species-specific feature. The hybrid tambacu is a very popular fish in Brazil, especially in the south and southeast regions and combines important characteristics for commercial fish farms, such as resistance to low temperatures during the tropical winter, similar to pacu, and faster growth, similar to tambaqui [1, 6]. Also, juvenile tambacu exhibits a total capacity for compensatory growth when submitted to short periods of food deprivation, followed by refeeding, a fact that could contribute to reducing production costs [42].

Although there are few studies showing the real potential of the hybrid for aquaculture production [42, 43], some of them have been done to better understand the genetics and molecular characteristics of this genotype. Recently, Gomes et al. used Next Generation Sequencing technologies for the analysis of muscle and skin transcriptome of *C. macropomum* and the hybrid tambacu, observed differences in the specificity and gene expression levels in both the muscle and skin of the hybrid tambacu and the parental tambaqui [44]. The authors demonstrated that in the muscle of tambaqui, up-regulated genes involved in muscle contraction, such as the myosin family, actin and myomesin, and catalytic protein genes. The hybrid tambacu muscle presented upregulated genes controlling oxidative stress, amino acid metabolism, the ubiquitin family, defense, and heat shock protein (HSP) family genes, involved in stress.

In our study, we can emphasize that the interaction of *jun* and *vegfa* genes with miR-206-3p (TC vs. TQ) and *lmo2* and *alas2* genes with miR-144-3p (TC vs. PC and TC vs. TQ) could provide an evolutionary advantage to the hybrid tambacu once these genes are controlling pathways related to blood vessel modulation, oxygen homeostasis, and oxidative metabolism. Taking this into account, the differential regulation of these miRNAs may represent an explanation of why the hybrid has a better adaptation from exposure to hypoxic environments [38, 45]. Besides, it could indicate an important function of miRNAs miR-206-3p and miR-144-3p in the control of the robustness and faster growth of the hybrid tambacu [46].

In addition, the most significant molecular function enriched by the targets of the up-regulated miRNAs in the tambacu muscle was transcriptional repressor activity. The inhibition of this function reinforces our hypothesis that these miRNAs could be related to the robustness and faster growth of the hybrid, since their activity may promote enhanced transcriptional activity, which combined with translation, is an essential process for muscle hypertrophy and growth [47].

## Conclusions

Using comparative global miRNA analysis in the hybrid tambacu and their parental genotypes *Piaractus mesopotamicus* and *Colossoma macropomum*, we demonstrated, for the first time, miRNAs differentially expressed in the skeletal muscle of the genotype comparison pairs (TC vs. PC, TC vs. TQ, and PC vs. TQ). Furthermore, we showed that the hybrid expressed miRNAs that control important molecular muscle markers with potential applications in aquaculture management systems. Our study also provides insight for further investigations involving the validation of these miRNAs and their signaling pathway components in muscle phenotype in these genotypes.

## Methods

### Experimental design, sample collection, Total RNA extraction, and small RNAs sequencing

Pure early juvenile fish *Colossoma macropomum*, *Piaractus mesopotamicus*, and hybrid tambacu (Juveniles, *n* = 5 per genotype) were obtained at the Aquaculture Center of UNESP - CAUNESP, and were maintained in aerated tanks in the Agribusiness Technology Agency (APTA), Presidente Prudente, Sao Paulo, until fish attained ~ 10 g. Fish were kept in a natural photoperiod (12 h light/dark), and water temperature around 28 °C. Dissolved oxygen and pH were monitored daily, and ammonia, nitrite, and nitrate were monitored weekly. Fish were fed with Guabi-Pirá 28 fish feed. Excess benzocaine (concentration over 250 mg/L) was applied to euthanize the animals. Afterward, body weight and total length were determined, and fast muscle samples were collected from the epaxial region near the dorsal fin. Muscle samples were then frozen in liquid nitrogen for molecular analysis.

Total RNA was isolated using TRIZOL (Invitrogen), following the manufacturer’s specifications. The total extracted RNA was treated with DNase (Life Technologies, USA), following the manufacturer’s specifications. All extracted RNA samples were analyzed by NanoVue Spectrophotometer (GE Healthcare) and Bioanalyzer (Agilent Technologies) for checking the total concentration and the RNA Integrity Number (RIN). The RNA extraction resulted in high quality RNA, and the RIN obtained was ≥8. Samples were submitted to the sequencing, comprehending 15 sequenced samples in total. The samples were shipped to LC Sciences (Houston, TX, USA) in dry ice, and the entire process of library preparation and sequencing by Illumina platform was carried out. Libraries preparation were carried out using the TruSeq Small RNA Library Preparation Kit, and samples were run in 50 cycle single-end Illumina HiSeq 2500 Fast Mode. After sequencing, the data were downloaded through a link provided by the company.

### MiRNA analysis

The processing pipeline consisted initially of analyzing the data regarding the read quality (Phred Score). For this purpose, the software FastQC (http://www.bioinformatics.babraham.ac.uk/projects/fastqc) version 0.10.1 was used. After obtaining the sequencing quality information, adapters were removed using the software Fastx-toolkit (http://hannonlab.cshl.edu/fastx_toolkit/index.html). Reads not containing adapters were kept in libraries. Reads that, after removing the adapters, presented less than 17 nucleotides in length were permanently excluded. A filter based on read-quality parameters using the Fastx-toolkit software was also carried out. In such way, 90% of the read content has a phred score of at least 30. Reads not satisfying these quality prerequisites were also permanently excluded. The libraries were then filtered by max length using a custom Perl script (Additional file 9), where reads longer than 26 nucleotides were discarded. The remaining reads were used to obtain an expression profile of known miRNAs, based on known miRNAs lists of *Danio rerio*, using data from mature and precursor miRNAs obtained from the miRBase database version 22 [4]. For return more alignment than other species, we selected *Danio rerio* as a reference for this study, which is considered a good biological model.

For the differential expression analysis of miRNAs, mature sequences were aligned against the precursor sequences using GMAP [48], using standard parameters, to create an annotation file in GFF format, making it possible to locate the mature sequences in the precursor sequences and differentiate alignments between the 5′ and 3′ miRNAs.

After building the annotation file, the filtered reads were aligned against the precursor references using Hisat2 [49] with standard parameters. The software featureCounts [50] was applied over the alignments to collect the read-count for each miRNA aligned against the precursor reference and matching the GFF annotation file. The counts were then analyzed to detect differentially expressed (DE) miRNAs using the Bioconductor/R package DESeq2 [51].

The three different genotypes received specific names regarding its popular names: PC - *Piaractus mesopotamicus* (**p**a**c**u), TQ - *Colossoma macropomum* (**t**amba**q**ui), and TC (hybrid **t**amba**c**u).

A priori*,* we performed contrasts between the hybrid and each parental species, always having the hybrid as a base of comparison (TC vs. PC and TC vs. TQ). A posteriori, for further understanding of the traits inherited by each parental, in terms of miRNA expression profiles, we also have run a contrast analysis between the two parental genotypes. Such a second contrast has pacu as a base of comparison (PC vs. TQ). In this way, three contrasts were obtained: TC vs. PC, TC vs. TQ, and PC vs. TQ (see Additional files 5, 6 and 7).

For considering DE characteristics, we established that miRNAs presenting log2FoldChange (log2FC) ≤ − 0.75 and ≥ 0.75, followed by adjusted *p*-value (padj) ≤ 0.05, were considered differentially expressed as downregulated and upregulated, respectively.

The list of DE miRNAs detected by DESeq2 analysis was applied to the drawing of Venn Diagrams to visualize the DE miRNA sharing characteristics of each genotype by using Venn 2.1.0 (http://bioinfogp.cnb.csic.es/tools/venny/). Venn diagrams were created in two ways, i.e., up-regulated miRNAs and down-regulated miRNAs.

The R package gplots was applied with the DE miRNAs list for obtain heatmaps, which gives us a graphical idea of the expression variations between all the contrasts.

Target genes were detected using MirTarBase [16] database and the results were applied to ontology analysis by using Enrichr [17] to retrieve information regarding pathways in which the target genes are involved. The MirTarBase search comprises only experimentally validated interactions classified as “strong evidence”.

Interaction networks were assembled using Cytoscape 3.7.1 [52] based on MirTarBase recovered data for a better observation of the interaction between DE miRNAs and their validated targets. Two sets of networks have been assembled: (i) a network containing validated interactions for *Homo sapiens*, *Mus musculus*, *Rattus norvegicus,* and *Danio rerio*, and (ii) a network containing only *Danio rerio* validated interactions.

All bioinformatics steps can be observed in a flowchart presented (Additional file 10).

## Supplementary Information

**Additional file 1.** Read number information. Read number throughout filtering process. Data showing the decrease number of reads according to filtering steps performed.

**Additional file 2. **GFF annotation. GFF file containing annotations of mature sequences over precursor sequences of *D. rerio*.

**Additional file 3. **FASTA file. FASTA file containing precursor sequences of *D. rerio*.

**Additional file 4.** Raw and normalized counts. Data showing the raw number of reads obtained from each miRNA detected in libraries, and normalized values.

**Additional file 5.** DESeq2 results. Total DESeq2 results and filtered DESeq2 results for TC vs PC contrasts.

**Additional file 6.** DESeq2 results. Total DESeq2 results and filtered DESeq2 results for TC vs TQ contrasts.

**Additional file 7.** DESeq2 results. Total DESeq2 results and filtered DESeq2 results for PC vs TQ contrasts.

**Additional file 8.** DESeq2 results. DESeq2 results showing only differential expressed miRNAs for TC vs PC, TC vs TQ and PC vs TQ contrasts.

**Additional file 9.** Perl script. Perl script used for filtering reads by size.

**Additional file 10.** Flowchart. Bioinformatics pipeline. (PPTX 50 kb)

## Data Availability

All raw and processed data have been deposited on the GEO dataset, under access number **GSE147532**.

## Abbreviations

PC: Pacu
TQ: Tambaqui
TC: Hybrid tambacu
DE miRNAs: Differentially expressed miRNAs
PCA: Principal Component Analysis
*vcana*: Versican a
*vegfa*: Vascular Endotelial Growth Factor A
*jun*: AP-1 Transcription factor Subunit
*cyb561d2*: Cytochrome B561 Family Member D2
*lmo2*: LIM Domain Only 2
*klfd*: Kruppel-like factor d
*gata2a*: GATA-binding protein 2
*meis1*: Meis Homeobox 1
*klf3*: Kruppel Like Factor 3
*alas2*: 5-aminolevulinate synthase 2
*hif1*: Hypoxia-Inducible Factor 1
COBEA: Brazilian College of Animal Experimentation
APTA: Agribusiness Technology Agency
RIN: RNA Integrity Number
