# Supplementary material for: Integrative microRNAome analysis of skeletal muscle of Colossoma macropomum (tambaqui), Piaractus mesopotamicus (pacu), and the hybrid tambacu, based on next-generation sequencing data
Source: BMC Genomics. 2021 Apr 6;22:237. doi: 10.1186/s12864-021-07513-5 (PMC8022549; doi:10.1186/s12864-021-07513-5)
Supplement: Supplementary file 1 — Additional file 1. Read number information. Read number throughout filtering process. Data showing the decrease number of reads according to filtering steps performed. [file 12864_2021_7513_MOESM1_ESM.docx]

Additional file 1: Read numbers throughout the filtering process. Raw (raw data after downloading), Clipped (remaining reads after removing adaptors and reads shorter than 17nt), Filtered (remaining reads after filtering by read quality) and Sized (remaining reads after removing sequences longer than 26nt).

| **Samples** | **Counts** | | | | **Overall Remaining**  **(%)** |
| --- | --- | --- | --- | --- | --- |
|  | **Raw** | **Clipped** | **Filtered** | **Sized** |  |
| PC10.fq | 10313969 | 6700040 | 6186043 | 5175685 | 50,181 |
| PC3.fq | 9556831 | 7037027 | 6515045 | 4862165 | 50,876 |
| PC4.fq | 12691214 | 10491945 | 9702000 | 5605631 | 44,169 |
| PC5.fq | 10515142 | 8095448 | 7429667 | 5423264 | 51,576 |
| PC8.fq | 14299559 | 10155641 | 9463554 | 9066029 | 63,401 |
| **Total** | **57376715** | **42480101** | **39296309** | **30132774** | **52,517** |
|  |  |  |  |  |  |
| TC3.fq | 10587226 | 8864783 | 8170938 | 7028534 | 66,387 |
| TC4.fq | 11376639 | 8900637 | 8245256 | 5294625 | 46,539 |
| TC5.fq | 12095957 | 9563897 | 8871556 | 6262094 | 51,770 |
| TC6.fq | 10629076 | 7982307 | 7382385 | 5159981 | 48,546 |
| TC8.fq | 10803236 | 7408988 | 6843503 | 5564997 | 51,512 |
| **Total** | **55492134** | **42720612** | **39513638** | **29310231** | **52,819** |
|  |  |  |  |  |  |
| TQ1.fq | 8813112 | 5776793 | 5325040 | 4269551 | 48,445 |
| TQ2.fq | 11393977 | 9392874 | 8686187 | 6552465 | 57,508 |
| TQ3.fq | 10699000 | 9050977 | 8388799 | 4449997 | 41,593 |
| TQ6.fq | 7126363 | 5258865 | 4919845 | 3451529 | 48,433 |
| TQ8.fq | 10026422 | 7132258 | 6586723 | 5314842 | 53,008 |
| **Total** | **48058874** | **36611767** | **33906594** | **24038384** | **50,019** |
