# Supplementary material for: Integrative microRNAome analysis of skeletal muscle of Colossoma macropomum (tambaqui), Piaractus mesopotamicus (pacu), and the hybrid tambacu, based on next-generation sequencing data
Source: BMC Genomics. 2021 Apr 6;22:237. doi: 10.1186/s12864-021-07513-5 (PMC8022549; doi:10.1186/s12864-021-07513-5)
Supplement: Supplementary file 10 — Additional file 10. Flowchart. Bioinformatics pipeline. (PPTX 50 kb) [file 12864_2021_7513_MOESM10_ESM.pptx]

## Slide 1
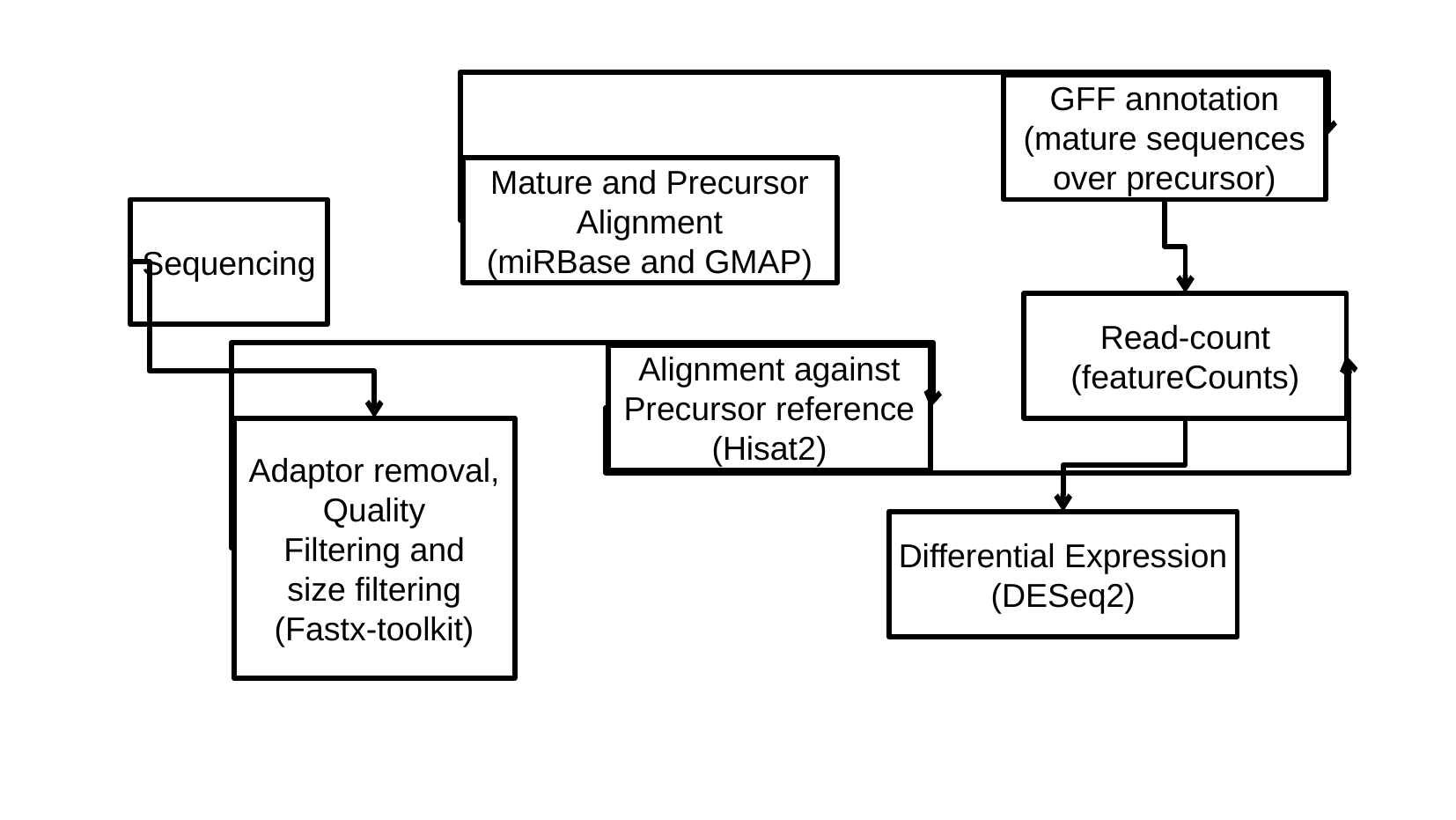

GFF annotation
(mature sequences
over precursor)
Mature and Precursor
Alignment
(miRBase and GMAP)
Sequencing
Read-count
(featureCounts)
Alignment against
Precursor reference
(Hisat2)
Adaptor removal,
Quality
Filtering and
size filtering
(Fastx-toolkit)
Differential Expression
(DESeq2)
